# Conserved Protective Mechanisms in Radiation and Genetically Attenuated *uis3(-)* and *uis4(-) Plasmodium* Sporozoites

**DOI:** 10.1371/journal.pone.0004480

**Published:** 2009-02-13

**Authors:** Kota Arun Kumar, Peter Baxter, Alice S. Tarun, Stefan H. I. Kappe, Victor Nussenzweig

**Affiliations:** 1 Micheal Hidelberg Division of Immunology, Department of Pathology, New York University School of Medicine, New York, New York, United States of America; 2 Department of Animal Sciences, School of Life Sciences, University of Hyderabad, Hyderabad, India; 3 Seattle Biomedical Research Institute, Seattle, Washington, United States of America; Queensland Institute of Medical Research, Australia

## Abstract

Immunization with radiation attenuated *Plasmodium* sporozoites (RAS) elicits sterile protective immunity against sporozoite challenge in murine models and in humans. Similarly to RAS, the genetically attenuated sporozoites (GAPs) named *uis3(-)*, *uis4(-)* and *P36p(-)* have arrested growth during the liver stage development, and generate a powerful protective immune response in mice. We compared the protective mechanisms in *P. yoelii* RAS, *uis3(-)* and *uis4(-)* in BALB/c mice. In RAS and GAPs, sterile immunity is only achieved after one or more booster injections. There were no differences in the immune responses to the circumsporozoite protein (CSP) generated by RAS and GAPs. To evaluate the role of non-CSP T-cell antigens we immunized antibody deficient, CSP-transgenic BALB/c mice, that are T cell tolerant to CSP, with *P. yoelii* RAS or with *uis3(-)* or *uis4(-)* GAPs, and challenged them with wild type sporozoites. In every instance the parasite liver stage burden was approximately 3 logs higher in antibody deficient CSP transgenic mice as compared to antibody deficient mice alone. We conclude that CSP is a powerful protective antigen in both RAS and GAPs viz., *uis3(-)* and *uis4(-)* and that the protective mechanisms are similar independently of the method of sporozoite attenuation.

## Introduction

To date only vaccines containing radiation attenuated sporozoites (RAS) consistently induce sterile immunity in rodents [1], monkeys [2] and humans [3]. Immunization of humans with *Plasmodium falciparum* sporozoites was accomplished by the bite of infected irradiated *Anopheles* mosquitoes, and after many booster injections a high degree of protection was obtained [3], [4]. The RAS protective immunity is mediated by antibodies to sporozoites and by effector CD4+ and CD8+ T cells against livers stages (exoerythrocytic stages or EEFs) [5]. The T cell protection is mediated in part by interferon-γ that promotes the production of NO in the infected hepatocyte and subsequent inhibition of the development of the early EEFs [6], [7], [8], [9], [10]. The antibodies are mostly or exclusively directed against the circumsporozoite protein (CSP) that covers the plasma membrane of the sporozoites [11]. These antibodies immobilize sporozoites [12], prevent their attachment to the host's hepatocytes [13], and inhibit infection. Since the sporozoites delivered by mosquito bite remain for a short time in the skin [14] and in the blood circulation [15], the titers of antibodies to CSP have to be very high to neutralize the infectivity of all incoming parasites. Therefore CD4+ and/or CD8+ effector T cells that recognize the infected hepatocytes are required to obtain sterile immunity in the murine models of pre-erythrocytic vaccines [16].

In addition to RAS, advances in reverse genetics led to the generation of the genetically attenuated parasites (GAP). The attenuated parasites named *uis3(-)*, *uis4(-)*, *P36p(-)* [17]–[21] were obtained by targeting sporozoite genes that are essential for completing the liver stage cycle. Recent studies using RAS immunization of CSP-transgenic BALB/c and C57BL/6 mice that are unable to make antibody responses showed that CSP is a powerful protective T cell antigen [22]. This apparent dominance was also documented recently in *Toxoplasma*. Among thousands of parasite proteins, only GRA6 generated a dominant CD8+ protective epitope [23]. It is known that T cells play a major role in GAP-mediated protection, but the corresponding antigens have not yet been identified [20], [24], [25]. This is highly desirable in order to establish correlates of protection following human vaccine trials. As a step in this direction, here we study the role of CSP in the immune responses of mice to *P. yoelii uis3(-)* and *uis4(-)* GAPs.

## Results

**Figure 1:**
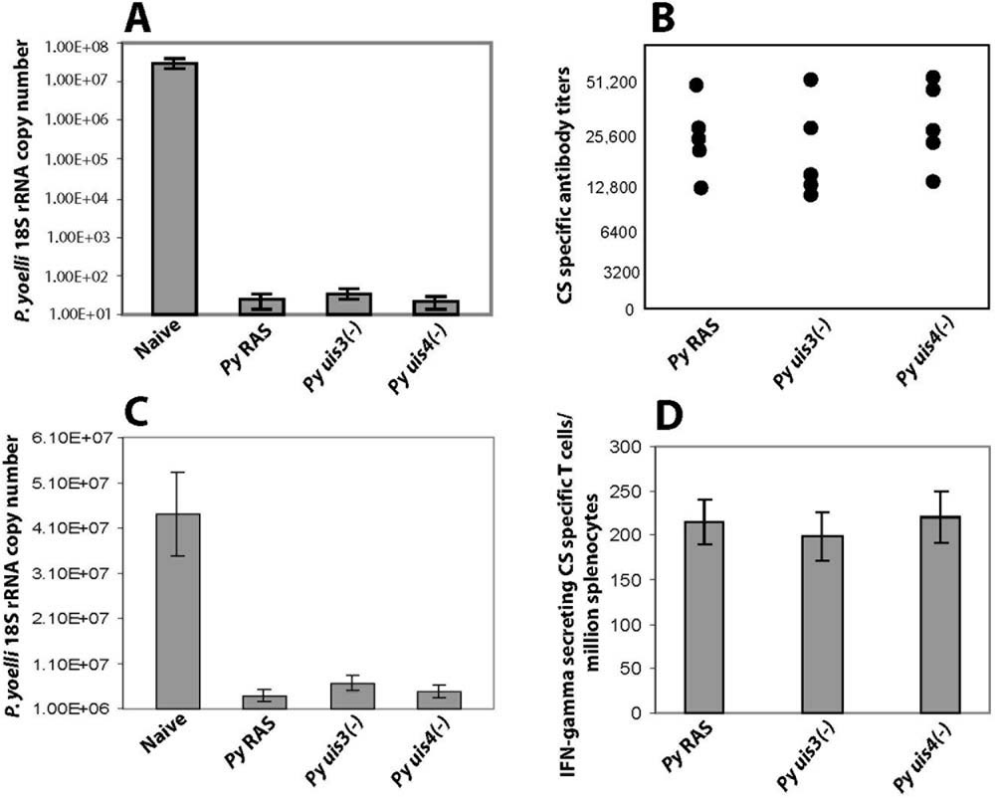
Protective immune responses are conserved in *P. yoelli* RAS and *uis3(-)*, *uis4(-)* GAPs. Comparative analysis of protective immune response in BALB/cAnN mice following priming and boosting with 1×10^5^ *P. yoelli* RAS or with *uis3(-)* or with *uis4(-)* GAPs. All mice were challenged with 1×10^4^ wild type infectious sporozoites and infected livers were isolated 42 hours post infection. (A) Liver stage burden in indicated groups of immunized mice were assessed by measuring the parasite specific 18S rRNA copy numbers by q-RT PCR. (B) CS-specific antibody response in indicated groups of immunized mice. (C) Liver stage burden in mice that received wild type *P. yoelli* sporozoites following neutralization with sera obtained from naïve or indicated groups of immunized mice. (D) IFN-gamma ELISPOT assay to quantify CS specific T cells from indicated groups of immunized mice. Results are expressed as mean±s.d of CS-specific CD8+ T cells obtained from 5 immunised mice per group. In (A) and (C) results are expressed as mean±s.d of 18S r RNA copy numbers from 5 mice per group.

Groups of BALB /c mice were primed and boosted 14 days later with 10^5^ *P. yoelii* RAS, or with 10^5^ *P. yoelii uis3(-)* or with *uis4(-)* GAPs. All animals were challenged a week later with 1×10^4^ wild type infectious sporozoites. The liver stage burdens were evaluated by q-RT PCR at 42 hours post infection when the EEFs are mature. We found that the levels of protection elicited by RAS or GAP vaccination were greater than 95% in all groups (Fig 1A). The anti-CSP antibody titers measured by ELISA against the repeat domain of the *P. yoelii* CSP ranged between 12,500 and 50,000 in all immunized mice (Fig 1B). To compare the neutralizing activity of the antibodies, *P. yoelii* salivary gland sporozoites were incubated with pooled immune sera from the respective immunized groups and injected into naïve mice and the liver stage burden was evaluated. In every instance the liver stage burdens were 8–9 fold lower than that of sporozoites inoculated with normal mouse serum (Fig 1C). The abundance of interferon-γ producing CD8+ T cells against the H2-K^d^ CTL epitope of CSP was evaluated by ex-vivo ELISPOT assay. The T cell responses amongst different groups of immunized mice were indistinguishable (Fig 1D). Therefore, irrespective of how the sporozoites were attenuated, the overall immune response of BALB/c mice directed against epitopes in CSP was very similar.

**Figure 2:**
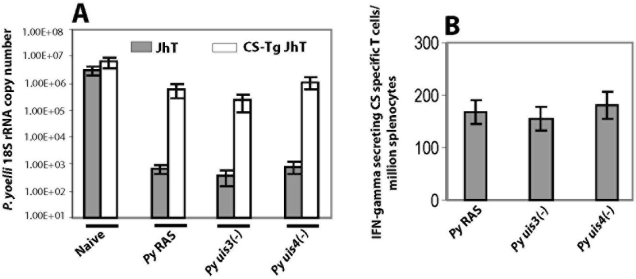
CS is a powerful protective T cell antigen in both *P. yoelli* RAS and *uis3(-)*, *uis4(-)* GAPs. BALB/c CS-Tg JhT (−/−) mice were primed and boosted with 1×10^5^ *P. yoelli* RAS or with *uis3(-)* or with *uis4(-)* GAPs. All immunized mice were challenged with 1×10^4^ wild type infectious sporozoites and infected livers were isolated 42 hours post infection (A) Liver stage burden in indicated groups of immunized mice were assessed by measuring the parasite specific 18S rRNA copy numbers by q-RT PCR. The results are expressed as mean±s.d of 18S rRNA copy numbers from 5 mice per group. (B) IFN-gamma ELISPOT assay to quantify CS specific T cells from indicated groups of JhT (−/−) immunized mice. Results are expressed as mean±s.d of CS-specific CD8+ T cells obtained from 5 immunized mice per group.

Next we compared the relative importance of CSP in the protective T cell responses to RAS and GAPs. For this purpose we used BALB/c mice that are both T-cell tolerant to CSP and cannot make antibodies [(CSP-transgenic, JhT (−/−)]. The mice were primed and boosted with RAS, or with *uis3(-) or uis4(-)* GAPs and challenged as above. We found that immunization with RAS or GAPs led to a reversal of protection by approximately 3 logs in CSP-transgenic, JhT (−/−) mice (Fig 2A), as compared to liver stage burden of an identically immunized JhT (−/−) mice. Further, RAS or GAP immunization in Jht (−/−) mice induced very similar numbers of interferon-γ producing T cells against the CD8+ T cell epitope of CSP (Fig 2B).

In order to obtain sterile immunity by RAS in mice at least one booster injections are generally required. In humans, complete protection against challenge was achieved only after many boosters over several months of *P. falciparum* infected and irradiated mosquitoes [4]. Similarly, GAP immunization with *P. yoelli uis3(-)* [20] or *P. berghei uis3(-)* [17] or *P. berghei uis4(-)* [18] only leads to complete protection after one or more booster injections. In contrast with these findings, it has been reported that BALB/cJ mice (Jackson labs) were completely protected by single dose immunizations with 50,000 *P. yoelii uis4(-)* sporozoites [20]. We repeated the same immunization protocol but used instead BALB/c mice bought from Taconic labs (BALB/cAnN), which have a different genetic background than BALB/cJ. As shown in Table 1 all immunized mice were infected after challenge, and there was a minimum delay in the pre-patent periods as compared to non-immunized controls. To evaluate the innate susceptibility of the two BALB/c substrains to *P. yoelii* sporozoites, the mice were iv injected with 1×10^6^ GFP-labeled parasites [26]. As assessed by microscopy or by fluorescence activated cell sorting of GFP+ infected hepatocytes, there were fewer numbers (10–15 fold) of liver stages in the BALB/cJ (Jackson) mice as compared to the BALB/cAnN (Taconic) (Sebastian A. Mikolajczak, Alice S. Tarun, Nelly Camargo, Mehdi Labaied, and Stefan HI Kappe, unpublished observations). Therefore, the previously observed sterile protection of BALB/cJ mice by a single immunization with *uis4(-)* [20] might be attributed to the decreased innate susceptibility of these mice to *P. yoelli* sporozoites. The underlying basis for the remarkable differences in the sporozoite infectivity for BALB/cJ and BALB/cAnN mice is under investigation.

**Table 1:** Single immunization of BALB/cAnN mice with *P. yoelli uis4(-)* GAP does not induce sterile immunity.

S.No	Group	Number of animals immunized/number of animals infected^*^ or challenged^#^ with 1×10^4^ infectious sporozoites	Prepatent period between days	Sterile immunity
1.	Naive	0/4^*^	2–2.5	NA
2.	Immunized with 5×10^4^ *P. yoelli uis4(-)* and challenged after 7 days	4/4**^#^**	2.5–3	None
3.	Immunized with 5×10^4^ *P.yoelli uis4(-)* and challenged after 14 days	4/4**^#^**	2.5–3	None

NA: Not Applicable.

## Discussion

Previous studies highlighted the central role of CD8+ T cells in protection of BALB/c mice after vaccination with *P. yoelii uis3(-)*, *uis4(-)* [20], and of C57BL/6 mice with *P. berghei uis3(-)*, *uis4(-)* [24] and *P36p(-)* [19] GAPS, but the respective protective antigen(s) have yet to be identified. The main finding of this paper is that the humoral and cellular responses of BALB/c mice to CSP by immunization with *P. yoelii* RAS, or *uis3(-)* or *uis4(-)* GAPs are indistinguishable. We also show that in these three experimental models CSP is a powerful protective T cell antigen. Indeed, there was a profound reversal of protection (by approximately 1,000 times) in immunized BALB/c mice that are CSP transgenic (T cell tolerant to CSP) and JhT (−/−) (unable to make antibodies).

Under natural conditions liver stages are very rare among the massive numbers of hepatocytes in the liver, and the development of the parasite is completed in a few days. Thus, effector CD4+ or CD8+ T cells have only a limited time to find and destroy the very few infected hepatocytes prior to the completion of development of the liver stages and the entry of thousands of merozoites in the blood circulation. Furthermore, sporozoites remain in the skin and in the blood circulation for a few hours, and neutralizing antibodies also have a limited period of time to prevent hepatocyte invasion.

What is then the explanation for the powerful antibody and T cell protective responses directed against CSP following RAS or GAS immunization? CSP enters both the class I and II pathways of T cell presentation, and the central repeat domain of CSP is a potent B cell immunogen. Antibodies to the repeats immobilize the parasite [12] and inhibit the attachment and subsequent infection of the liver cells [13]. CSP is synthesized in large amounts by salivary gland sporozoites [27], and continues to be transcribed in the liver stages [28]. It is continuously shed by sporozoites during their migration through cells from the site of injection until they reach hepatocytes. Therefore, CSP enters not only the blood circulation but is also released into the cytoplasm of traversed cells where it can be processed and presented to T cells. The C-terminus of CSP contains a very powerful H2-Kd restricted CD8+ epitope [29], and a “promiscuous” or “HLA degenerate” CD4+ epitope [30].

Although our studies did not include immunization of mice with another GAP, *P36p(-)*, and is limited to BALB/c mice, on the basis of the present findings, it is likely that CSP will also play a major role in protection in those additional experimental models. In fact, CSP is also a protective antigen not only in mice but also in humans. To date, the only vaccine that has been proven to be effective in naïve human volunteers and in endemic areas is RTS,S that contains the repeats and the C-terminus of the CSP of *P. falciparum* [31]. We emphasize, however, that CSP is not the sole protective T cell immunogen present in RAS or GAPs. As shown elsewhere, after priming and two booster injections with RAS transgenic CSP mice can be fully protected against challenge [22]. Consistent with these observations was a recent study that demonstrated that sterile protection against malaria is independent of immune responses to the circumsporozoite protein [32]. In this study, *P.berghei* RAS immunized mice were challenged with a transgenic *P.berghei* line expressing heterologous CSP from *P. falciparum* [*PfCS*]. Complete sterile protection of *P.berghei* RAS immunized mice suggested that protection is independent of immune targets from the immunodominant CSP of *P.berghei*. This study together with ours [22], provide evidence for the presence of hitherto uncharacterized antigens that can be targeted to induce sterile immunity against malaria. However, if we consider the stringent requirements for inhibition of sporozoite infectivity that we mentioned above, we argue that the parasite contains only a few effective T and B cell antigens. Our findings suggest that these few non-CS antigens are shared between RAS and GAPs.

## Materials and Methods

### Growing and harvesting *P. yoelli uis3(-)*, *uis4(-)* and wild type sporozoites

Female Swiss Webster mice were infected with blood stages of *Py uis3(-)*, *uis4(-)* and wild type parasites to initiate a gametocyte infection. Mice having 2–3% gametocytes were used for feeding female *Anopheles* mosquitoes. The Institutional Animal Care and Use Committee of New York University approved all experimental procedures involving mice. The mosquitoes were dissected on day 8 to analyze their midgut infectivity. On day 14, the salivary glands were dissected to harvest infective sporozoites. The *uis3(-)*, *uis4(-)* and wild type sporozoites were used for immunization experiments. Radiation attenuation of the wild type sporozoites were carried out by exposing them to 10,000 rads (cesium radiation source) for 15 minutes.

### Immunisation of BALB/c and CSP-transgenic JhT (−/−) mice with RAS and GAPs

To study the comparative immune responses against *P.yoelli* RAS and GAPs, BALB/cAnN (Taconic) mice were primed and boosted with 1×10^5^ sporozoites. Similar immunization regimens were performed in CS-Tg mice that are obtained in JhT (−/−) background (antibody deficient mice) to evaluate the relevance of pre-erythrocytic T cell antigens other than CS. Seven days after final immunization, the mice were challenged with 1×10^4^ wild type infectious sporozoites. Blood, spleen and liver samples were harvested to analyze the anti-CS antibody levels, CS-specific T cells responses and liver stage burden in all challenged mice. To monitor sterile immunity after single immunization two groups of BALB/cAnN mice were immunized with 5×10^4^ *Py uis4(-)* sporozoites and challenged on either day 7 or day 14 with 1×10^4^ wild type *P.yoelli* sporozoites. Blood smears were made from day 2 post infection to monitor the appearance of blood stage parasites.

### IFN-gamma ELISPOT assay

To quantify the CS-specific T cell responses induced following immunization with RAS and GAP, splenocytes were obtained from all immunized mice and assayed by ELISPOT following the method described earlier [33]. For these assays we used MHC compatible A20.2J cells coated with SYVPSAEQI peptide that stimulate CS-specific CD8+ T cells to secrete IFN-gamma.

### ELISA

Immune sera were obtained from different immunized groups of mice and were assayed for CS specific antibody titers. Recombinant GST fusion protein containing the B cell epitope of circumsporozoite protein was used as coating antigen in ELISA [22]. ELISA was performed essentially as described earlier [34].

### Sporozoite Neutralization assay

To assess the ability of immune sera to neutralize the infectivity of the sporozoites, 1×10^5^ wild type *P. yoelli* sporozoites were incubated in the pooled immune sera obtained from different groups of immunized BALB/c mice. The sporozoites were incubated for 35 minutes at 37°C. Following neutralization 2×10^4^ sporozoites were injected intravenously into each naïve mouse. Quantification of *P. yoelli* 18S rRNA copy number was performed essentially as described earlier [35] except that a double stranded-DNA specific iQ SYBR Green supermix was used to detect the PCR product. The amounts of parasite-derived 18S rRNA copies were determined from a standard curve, generated with known amounts of 18S rRNA plasmid.

## References

[1] Nussenzweig RS, Vanderberg J, Most H, Orton C (1967). Protective immunity produced by the injection of x-irradiated sporozoites of *Plasmodium berghei*.. Nature.

[2] Gwadz RW, Cochrane AH, Nussenzweig V, Nussenzweig RS (1979). Preliminary studies on vaccination of rhesus monkeys with irradiated sporozoites of *Plasmodium knowlesi* and characterization of surface antigens of these parasites.. Bull World Health Organ.

[3] Clyde DF, Most H, McCarthy VC, Vanderberg JP (1973). Immunization of man against sporozoite-induced *falciparum* malaria.. Am J Med Sci.

[4] Hoffman SL, Goh LM, Luke TC, Schneider I, Le TP (2002). Protection of humans against malaria by immunization with radiation-attenuated *Plasmodium falciparum* sporozoites.. J Infect Dis.

[5] Rodrigues M, Nussenzweig RS, Zavala F (1993). The relative contribution of antibodies, CD4+ and CD8+ T cells to sporozoite-induced protection against malaria.. Immunol.

[6] Schofield L, Ferreira A, Altszuler R, Nussenzweig V, Nussenzweig RS (1987). Interferon gamma inhibits the intrahepatic development of malaria parasites invitro.. J Immunol.

[7] Ferreira A, Schofield L, Enea V, Schellekens H, van der Meide P (1986). Inhibition of development of exoerythrocytic forms of malaria parasites by gamma-interferon.. Science.

[8] Doolan DL, Hoffman SL (2000). Complexity of protective immunity against liver stages of Malaria.. J Immunol.

[9] Klotz FW, Scheller LF, Sequin MC, Kumar N, Marletta MA (1995). Co-localisation of inducible-nitric oxide synthase and *Plasmodium berghei* in hepatocytes from rats immunized with irradiated sporozoites.. J Immunol.

[10] Chakravarthy S, Baldeviano GC, Overstreet MG, Zavala F (2008). Effector CD8+ T lymphocytes against malaria liver stages do not require IFN-γ for anti-parasite activity.. Infect Immun.

[11] Nussenzweig V, Nussenzweig RS (1989). Rationale for the development of an engineered sporozoite malaria vaccine.. Adv Immunol.

[12] Stewart MJ, Nawrot RJ, Schulman S, Vanderberg JP (1986). *Plasmodium berghei* sporozoite invasion is blocked in vitro by sporozoite-immobilizing antibodies.. Infect Immun.

[13] Hollingdale MR, Zavala F, Nussenzweig RS, Nussenzweig V (1982). Antibodies to the protective antigen of *Plasmodium berghei* sporozoites prevent entry into cultured cells.. J Immunol.

[14] Amino R, Thiberge S, Martin B, Celli S, Shorte S (2006). Quantitative imaging of *Plasmodium* transmission from mosquito to mammal.. Nat Med.

[15] Vanderberg JP, Frevert U (2005). Intravital microscopy demonstrated antibody mediated immobilization of *Plasmodium berghei* sporozoite infection of the liver.. PLoS Biol.

[16] Tsuji M, Zavala F (2003). T cells as mediators of protective immunity against liver stages of *Plasmodium*.. Trends Parasitol.

[17] Mueller AK, Labaied M, Kappe SH, Matuschewski K (2005). Genetically modified *Plasmodium* parasites as a protective experimental malaria vaccine.. Nature.

[18] Mueller AK, Camargo N, Kaiser K, Andorfer C, Frevert U (2005). *Plasmodium* liver stage developmental arrest by depletion of a protein at the parasite-host interface.. Proc Natl Acad Sci USA.

[19] van Dijk MR, Douradinha B, Franke-Fayard B, Heussler V, van Dooren MW (2005). Genetically attenuated, *P36p*-deficient malarial sporozoites induce protective immunity and apoptosis of infected liver cells.. Proc Natl Acad Sci U S A.

[20] Tarun AS, Dumpit RF, Camargo N, Labaied M, Liu P (2007). Protracted sterile protection with *Plasmodium yoelii* pre-erythrocytic genetically attenuated parasite malaria vaccines is independent of significant liver-stage persistence and is mediated by CD8+ T cells.. J Infect Dis.

[21] Douradinha B, van Dijk MR, Ataide R, van Gemert GJ, Thompson J (2007). Genetically attenuated P36p-deficient *Plasmodium berghei* sporozoites confer long-lasting and partial cross-species protection.. Int J Parasitol.

[22] Kumar KA, Sano G, Boscardin S, Nussenzweig RS, Nussenzweig MC (2006). The circumsporozoite protein is an immunodominant protective antigen in irradiated sporozoites.. Nature.

[23] Blanchard N, Gonzalez F, Schaffer M, Joncker NT, Cheng T (2008). Immunodominant, protective response to the parasite *Toxoplasma gondii* requires antigen processing in the endoplasmic reticulum.. Nat Immunol.

[24] Jobe O, Lumsden J, Mueller AK, Williams J, Silva-Rivera H (2007). Genetically attenuated *Plasmodium berghei* liver stages induce sterile protracted protection that is mediated by major histocompatibility complex Class I-dependent interferon-gamma-producing CD8+ T cells.. J Infect Dis.

[25] Mueller AK, Deckert M, Heiss K, Goetz K, Matuschewski K (2007). Genetically attenuated *Plasmodium berghei* liver stages persists and elicit sterile protection primarily via CD8 T cells.. Am J Pathol.

[26] Tarun AS, Baer K, Dumpit RF, Gray S, Lejarcegui N (2006). Quantitative isolation and invivo imaging of malaria parasite liver stages.. Int J Parasitol.

[27] Yoshida N, Potocnjak P, Nussenzweig V, Nussenzweig RS (1981). Biosynthesis of Pb44, the protective antigen of sporozoites of *Plasmodium berghei*.. J Exp Med.

[28] Zhou Y, Ramachandran V, Kumar KA, Westenberger S, Refour P (2008). Evidence-based annotation of the malaria parasite's genome using comparative expression profiling.. PLoS ONE.

[29] Rodrigues MM, Cordey AS, Arreaza G, Corradin G, Romero P (1991). CD8+ cytolytic T cell clones derived against the *Plasmodium yoelii* circumsporozoite protein protect against malaria.. Int Immunol.

[30] Sinigaglia F, Guttinger M, Kilgus J, Doran DM, Matile H (1988). A malaria T-cell epitope recognized in association with most mouse and human MHC class II molecules.. Nature.

[31] Alonso PL, Sacarlal J, Aponte JJ, Leach A, Macete E (2005). Duration of protection with RTS,S/AS02A malaria vaccine in prevention of *Plasmodium falciparum* disease in Mozambican children: single-blind extended follow-up of a randomised controlled trial.. Lancet.

[32] Gruner AC, Mauduit M, Tewari R, Romero JF, Depiny N (2007). Sterile protection against malaria is independent of immune responses to the circumsporozoite protein.. PLoS ONE.

[33] Carvalho LH, Hafalla JC, Zavala F (2001). ELISPOT assay to measure antigen specific murine CD8+T cell responses.. J Immunol Methods.

[34] Kumar KA, Oliviera GA, Edelman R, Nardin EH, Nussenzweig V (2004). Quantitative *Plasmodium* sporozoite neutralization assay (TSNA).. J Immunol Methods.

[35] Bruña-Romero O, Hafalla JC, González-Aseguinolaza G, Sano G, Tsuji M (2001). Detection of malaria liver-stages in mice infected through the bite of a single Anopheles mosquito using a highly sensitive real-time PCR.. Int J Parasitol.

